# SARS-CoV-2 journey: from alpha variant to omicron and its sub-variants

**DOI:** 10.1007/s15010-024-02223-y

**Published:** 2024-03-30

**Authors:** Dima Hattab, Mumen F. A. Amer, Zina M. Al-Alami, Athirah Bakhtiar

**Affiliations:** 1 School of Pharmacy, The University of Jordan, Queen Rania Street, Amman, Jordan; 2 Faculty of Pharmacy, Applied Science Private University, Amman, Jordan; 3 Department of Basic Medical Sciences, Faculty of Allied Medical Sciences, Al-Ahliyya Amman University, Amman, Jordan; 4 School of Pharmacy, Monash University Malaysia, Jalan Lagoon Selatan, 47500 Bandar Sunway, Selangor Malaysia

**Keywords:** Genetic surveillance, Monoclonal antibodies, Neutralizing antibodies, SARS-COV-2, Spike mutations, Viral virulence

## Abstract

The COVID-19 pandemic has affected hundreds of millions of individuals and caused more than six million deaths. The prolonged pandemic duration and the continual inter-individual transmissibility have contributed to the emergence of a wide variety of SARS-CoV-2 variants. Genomic surveillance and phylogenetic studies have shown that substantial mutations in crucial supersites of spike glycoprotein modulate the binding affinity of the evolved SARS-COV-2 lineages to ACE2 receptors and modify the binding of spike protein with neutralizing antibodies. The immunological spike mutations have been associated with differential transmissibility, infectivity, and therapeutic efficacy of the vaccines and the immunological therapies among the new variants. This review highlights the diverse genetic mutations assimilated in various SARS-CoV-2 variants. The implications of the acquired mutations related to viral transmission, infectivity, and COVID-19 severity are discussed. This review also addresses the effectiveness of human neutralizing antibodies induced by SARS-CoV-2 infection or immunization and the therapeutic antibodies against the ascended variants.

## Introduction

**Fig. 1:**
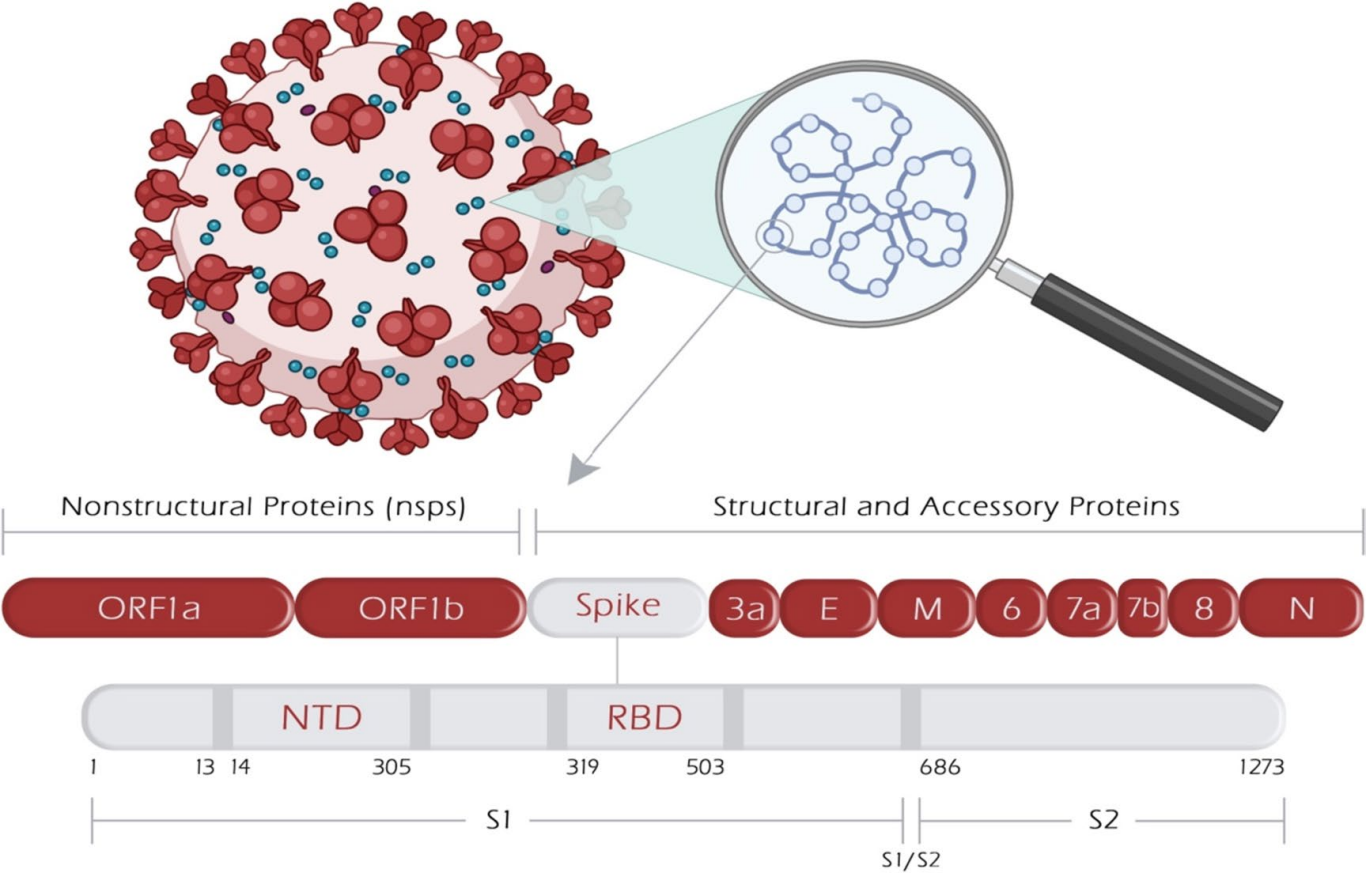
Schematic illustration of SARS-CoV-2 genome architecture showing protein-encoding regions. ORF1a and ORF1b encode nonstructural proteins. Four structural proteins (S, E, M, and N) and accessory proteins (designated by numbers) are encoded. E, envelope protein; M, membrane protein; N, nucleocapsid protein; NTD-N-terminal domain; ORF, open reading frame; RBD, receptor-binding protein

In recent decades, two significant coronavirus pandemics have emerged: severe acute respiratory syndrome (SARS, 2002–2003) and Middle East Respiratory Syndrome (MERS, 2012) [1]. More recently, the outbreak of a novel coronavirus, SARS-CoV-2, led to the devastating global pandemic known as Coronavirus Disease 2019 (COVID-19) [2]. Coronaviruses are RNA viruses characterized by distinctive crown-like surface proteins (Fig. 1). The viral RNA is positive-sense, single-stranded, and polyadenylated, enclosed within a capsid containing the nucleocapsid protein (N). The capsid is surrounded by an outer membrane composed of the membrane proteins (M), the envelope protein (E), and the spike protein (S) [3, 4]. In terms of size, the genetic material of coronaviruses ranks as one of the most extensive among known viral RNAs, encompassing approximately 30,000 nucleotides and encoding roughly 9,860 amino acids (aa). The SARS-CoV-2 genome expresses structural proteins (E, M, N, and S), non-structural proteins, and accessory proteins [5]. The spike protein consists of 1,273 amino acids, which make up the S1 (amino acids 14–685) and S2 subunits (amino acids 686–1273). The N-terminal domain (NTD) (amino acids 14–305) and the receptor-binding domain (RBD) (amino acids 319–541) are localized within the S1 subunit. The RBD contains the receptor-binding motif (RBM), which extends over 90 amino acids (amino acids 437–503) [6].

The interaction between the SARS-CoV-2 spike protein and human angiotensin-converting enzyme 2 (ACE2) receptors is crucial for viral replication and infectivity [7]. The RBD, particularly RBM, binds to ACE2 receptors, which triggers conformational changes in the S2 subunit, enhancing virus internalization [8]. As the virus continues to spread, it undergoes genetic changes, leading to antigenic drift within the genome of SARS-CoV-2. From 2019 to 2021, a multitude of genomic mutations surfaced, leading to variations in transmissibility, COVID-19 pathogenicity, and immunological resistance of COVID-19, regionally and globally. SARS-CoV-2 variants, such as B.1.1.7, B.1.351, P.1, B.1.617.2, and B.1.1.529 lineages, have been identified as variants of concern (VOC) for exhibiting increased transmissibility, disease severity, and hospitalization rates. Other variants such as B.1.526 and its sub-lineages, B.1.617.1 and B.1.617.3, were initially categorized as variants of interest (VOI). Over time, many VOCs and VOIs have seen a relative decline in their public health significance and are now classified as variants being monitored (VBM) [9, 10].

The diversification of genetic mutations within SARS-CoV-2 surpasses that of other viruses, with a notable accumulation of vital mutations in the NTD or receptor-binding domain (RBD) of the spike glycoprotein. Notably, the N501Y mutation is present in B.1.1.7, B.1.351, and P.1 variants, whereas E484K and RBD mutations are found in B.1.351, P.1, and B.1.526 lineages [11–13]. Additionally, variants like B.1.427/B.1.429, B.1.526-L452, and B.1.617 bear the L452R spike mutation [14].

SARS-CoV-2 variants carrying the E484K mutation exhibit resistance to neutralizing antibodies produced through natural infection or vaccination, resulting in diminished effectiveness of the majority of immunological therapeutics authorized by the Food and Drug Administration (FDA) [15, 16]. The N501Y mutation within the RBD of B.1.1.7, B.1.351, and P.1 variants enhances the binding affinity of spike protein to ACE2 receptors, resulting in increased transmissibility compared to non-variant lineages [17, 18]. Variants with L452R and E484Q spike mutations modestly enhance viral entry, infectivity, and immune evasion [14]. The Omicron variant and its sub-lineages are the most recent additions to the emerging variants. Omicron exhibits a unique genomic signature distinct from the original Wuhan SARS-CoV-2 strain [19]. Despite its notable surge in transmissibility and infectivity among the human population, Omicron appears to be associated with diminished COVID-19 pathogenicity and a reduced need for hospitalization in infected patients [18]. Notably, the emergence of Omicron and the subsequent recombinant variant-defining mutations have resulted in a complete loss of neutralizing responses of monoclonal antibodies (mAbs) against the variant.

This review article aims to shed light on the diverse genetic mutations in various SARS-CoV-2 phenotypes, beginning with the first mutated G614D variant and culminating in the newly emerged recombinant SARS-CoV-2 and its sub-variants. This review explores the implications of these mutations concerning viral transmission, infectivity, COVID-19 severity, the effectiveness of human neutralizing antibodies induced by SARS-CoV-2 infection or immunization, and therapeutic antibodies targeting the evolved phenotypes.

## The genomic phenotypes of different SARS-CoV-2 variants

The complete genetic sequence of SARS-CoV-2 was published early in 2020 [20]. One of its earliest genomic evolution was identified as D614G substitution [21, 22]. This substitution is present in all SARS-CoV-2 B.1 lineages [11]. Additionally, the B.1.1.7 lineage was the first SARS-COV-2 variant designated as VOC in the United Kingdom (UK) [9]. It is characterized by 17 mutations, of which eight mutations occur in the spike protein, namely Y144 del, H69-V70 del, N501Y, A570D, P681H, T716I, S982A, and D1118H (Table 1). In addition to the D614G substitution, several other key mutations in the spike glycoprotein are worth highlighting. These mutations include the N501Y mutation in RBD, the P681H mutation adjacent to furin cleavage supersite, and ΔH69/ΔV70 and Y144 deletions in NTD [23]. N501Y mutation is associated with the capability of the virus to attach to its receptors [24]. P681H mutation and ΔH69/ΔV70 deletion are linked with virus transmissibility and infectivity; ΔH69/ΔV70 deletion may also contribute to immunity escape [12]. Another significant variant, B.1.351, was identified in South Africa and classified as a VOC due to its increased transmissibility and global spread [25]. This variant carries nine critical spike mutations: four in the NTD (242–244 del, R246I), three in the RBD (N501Y, E484K, and K417N), and one proximal to the furin cleavage site (A701V) [26–28].

**Table 1:**
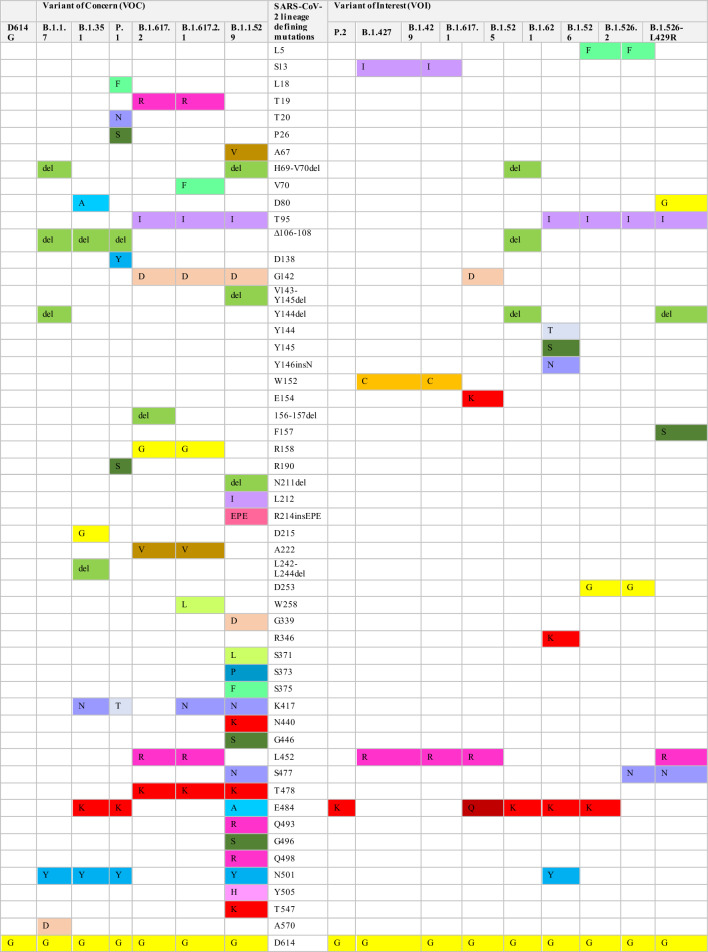

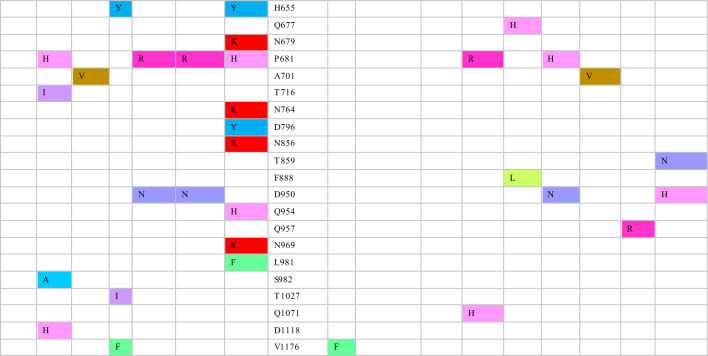
The defining mutations in the spike protein of SARS-CoV-2 in various VOC and VOI

The P.1 lineage is a VOC derived from the B.1.1.28 strain and was initially identified in Brazil [13, 29]. P.1 exhibits a distinct constellation of genomic mutations compared to the two previously mentioned lineages. Among the notable mutations in P.1 is N501Y, which is shared with B.1.1.7, B.1.351, and P.1 lineages. Additionally, P.1 is marked by convergent RBD mutations, such as L18F and E484K, in comparison with the B.1.351 lineage. P.1 also carries a unique K417T substitution instead of the K417N mutation found in B.1.351. Other distinctive spike mutations are observed in P.1, including T20N, P26S, D138Y, and R190S in the NTD, as well as H655Y, which is situated close to the furin cleavage site [30]. Another SARS-CoV-2 phenotype known as P.2 was isolated in Brazil and Japan. The whole genome sequence of P.2 closely resembles that of P.1 and B.1.351 variants, all of which harbor the E484K spike mutation [14, 31, 32]. The B.1.427/B.1.429 lineage, initially identified in California, USA, is characterized by the L452R spike mutation. Additionally, this lineage bears distinct defining mutations in the spike glycoprotein, namely S13I and W152C, alongside the L452R mutation. Other coding and non-coding mutations are found in the non-spike region (Table 1) [33].

Over time, various SARS-CoV-2 VOIs emerged worldwide [9]. The VOI strain identified as B.1.525 in the UK carries a cluster of mutations including ∆H69-∆V70 deletion (found in B.1.1.7), ∆106–108 deletion (present in B.1.1.7, P.1 and B.1.351), and E484K (observed in B.1.351, P.1 and P.2 strains). Distinctive spike mutations in B.1.525 include ∆144 deletions, F888L, and Q677H (Table 1) [34]. Another VOI that emerged in New York, USA, is called B.1.526, which is characterized by E484K substitution in RBD of the spike protein. Phylogenetic analysis revealed three sub-lineages within the B.1.526 phenotype: B.1.526, B.1.526.2, and B.1.526-L452R. These sub-lineages differ in the crucial spike mutations. The B.1.526 variant carries the E484K mutation, while the B.1.526.2 lineage harbors the S477N mutation. In contrast, the characteristic mutation in the B.1.526-L452R phenotype is L452R. Several spike mutations, such as L5F, T95I, and D253G, are found in B.1.526 and B.1.526.2 but not in B.1.526-L452R phenotypes. Furthermore, A701V is present in the B.1.526 spike protein, whereas Q957R is observed in the B.1.526.2 lineage [35, 36].

Critical mutations found in previous VOC are also found in B.1.621 VOI, including N501Y and E484K in RBD, and P681H near the furin cleavage site. Additionally, mutations namely T95I, Y144T, and Y145S substitutions, and a 146N insertion in the NTD have been found unique in the B.1.621 spike proteins (Table 1). The 146N insertion in NTD is linked to conformational changes of RBD, which affects the binding affinity of the virus to ACE2 receptors [37].

The discovery of the B.1.617 phenotype and its sub-lineages, B.1.617.1, B.1.617.2, and B.1.617.3, was initially made in India. They share several spike mutations, including T19R, G142D, or D950N and, notably, L452R (Table 1). The L452R substitution enhances spike-ACE2 binding and fusogenecity of viral S2 subunits in host cells [38–41]. B.1.617.1 and B.1.617.3 share E484Q substitution, whereas B.1.617.2 (Delta) is a VOC with a range of mutations in both NTD and RBD (L452R and E484Q) and at the region close to the furin cleavage site (P681R) (Table 1) [42, 43]. These mutations are responsible for the quick spread and evasion of the immune system [9]. Several Delta Plus sub-lineages derived from B.1.617.2 showcase additional genomic mutations, such as spike mutations like V70F, K417N, and W258L, and various non-spike mutations (Table 1) [44–46].

The variant identified in South Africa in November 2021 called Omicron BA.1 is recognized as VOC [9]. It quickly became predominant, surpassing Delta and Delta Plus variants within two months [47]. The variant carries more than 30 defining spike mutations, which include deletion of 69–70, T95I, K417N, S477N, T478K, D614G, H655Y, and P681H. Additionally, it has around 23 mutations unique to Omicron BA.1 (Table 1) [17]. Within a brief period, various Omicron sub-variants surfaced, such as BA.2, BA.3, BA.4, and BA.5. These sub-variants exhibit genetic resemblances to Omicron BA.1 and are linked to heightened transmissibility, contributing to an increase in global cases. Notably, each sub-variant bears its unique mutations and characteristics. Omicron BA.1 is characterized by notable mutations, namely T19I, L24 del, P25 del, P26 del, A27S, V213G, T376A and R408S (Table 2). Some derivatives of BA.2, such as BA.2.11 and BA.2.12.1, bear L452R spike mutation, whereas others exhibit L452Q and S704L substitutions [18]. Spike mutations in Omicron BA.4 and BA.5 sub-variants are akin to those found in Omicron BA.2, yet they differ in certain mutations like 69-70del, L452R, F486V, and R493Q. The main distinguishing feature between BA.4 and BA.5 lies in their non-spike mutations [48].

**Table 2:**
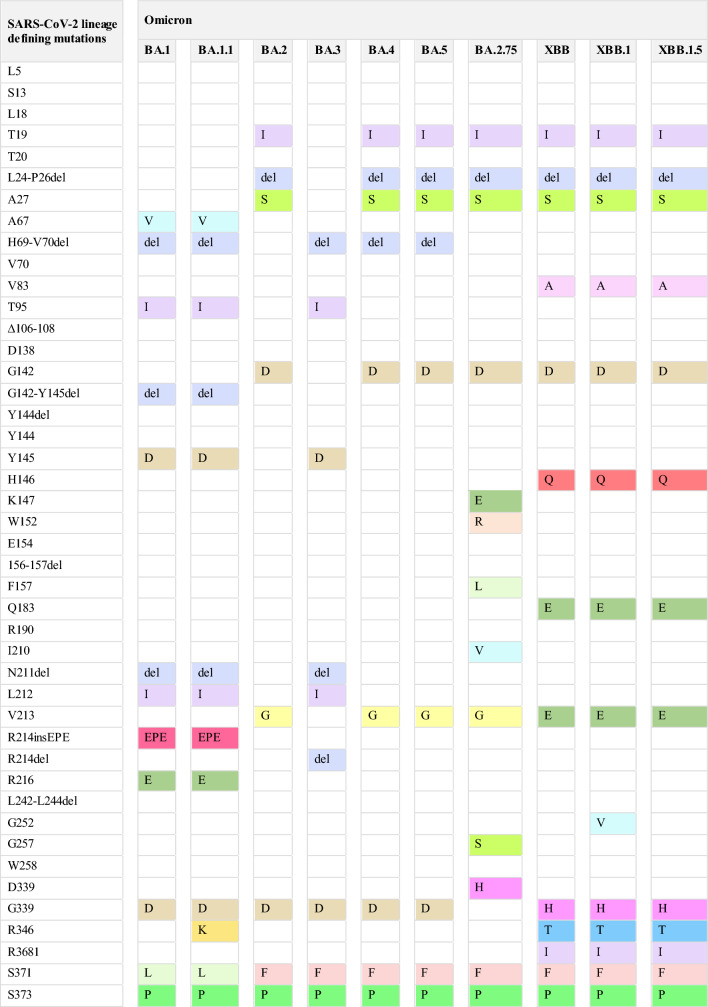

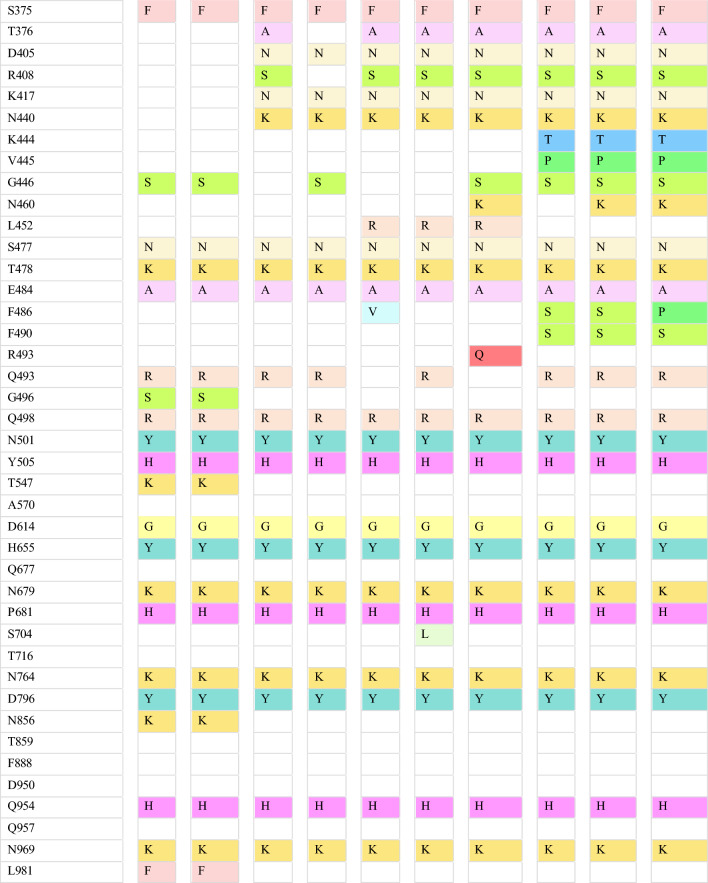
SARS-CoV-2 defining mutations in the spike protein for Omicron BA.1 and its sub-lineages

Genetic surveillance of the newly emerged sub-lineage, BA.2.75, revealed nine mutations in NTD and RBD, viz., K147E, W152R, F157L, I210V, G257S, and D339H, G446S, N460K, and R493Q (Table 2) [49]. Unlike the original Omicron and some of its sub-variants (BA.4 and BA.5), Omicron BA.2 and its descendant sub-lineage, BA.2.75, do not exhibit the defining spike deletion found in Omicron BA.1. Consequently, these sub-variants cannot be easily identified by PCR tests [50].

The emergence of recombinant SARS-CoV-2 variants since the beginning of 2022 has resulted in a surge of COVID-19 cases from time to time. Among the most important recombinant variants identified are XBB and its subvariants (XBB.1, XBB.1.5. XBB.1.16, XBB.1.9.1, XBB.1.9.2, and XBB.2.3) [51]. XBB is a recombinant SARS-CoV-2 derived from two Omicron BA.2 subvariants, namely BA.2.75 and BA.2.10.1. Compared with Omicron BA.2, BXX spike protein has an additional 14 mutations, of which nine are found in the RBD region (R346T, N460K, F486S, F490S, G446S, K444T, and V455P) (Table 2). XBB.1 carries the same mutation found in the XBB variant with an additional G252V mutation. The subsequent derivative, XBB.1.5 subvariant, shows an extra F486P mutation in addition to the mutations it shared with XBB.1 [52]. Notably, some of the critical spike sites, 346, 444, 452, 460, and 486, are convergently substituted in most of these variants [53]. It is presumed that these convergent mutations emerged as a result of selective pressure due to the therapeutics targeting spike protein (anti-spike mAbs, vaccine, and infection-induced antibodies) [54]. A BA.5. derivative called BQ.1.1 variant first emerged in November 2022, harboring five new convergent mutation substitutions, namely R346T, K444T, L452R, N460K, and F486V [9].

## Viral virulence and disease pathogenicity impaction of SARS-CoV-2 acquired mutations

**Fig. 2:**
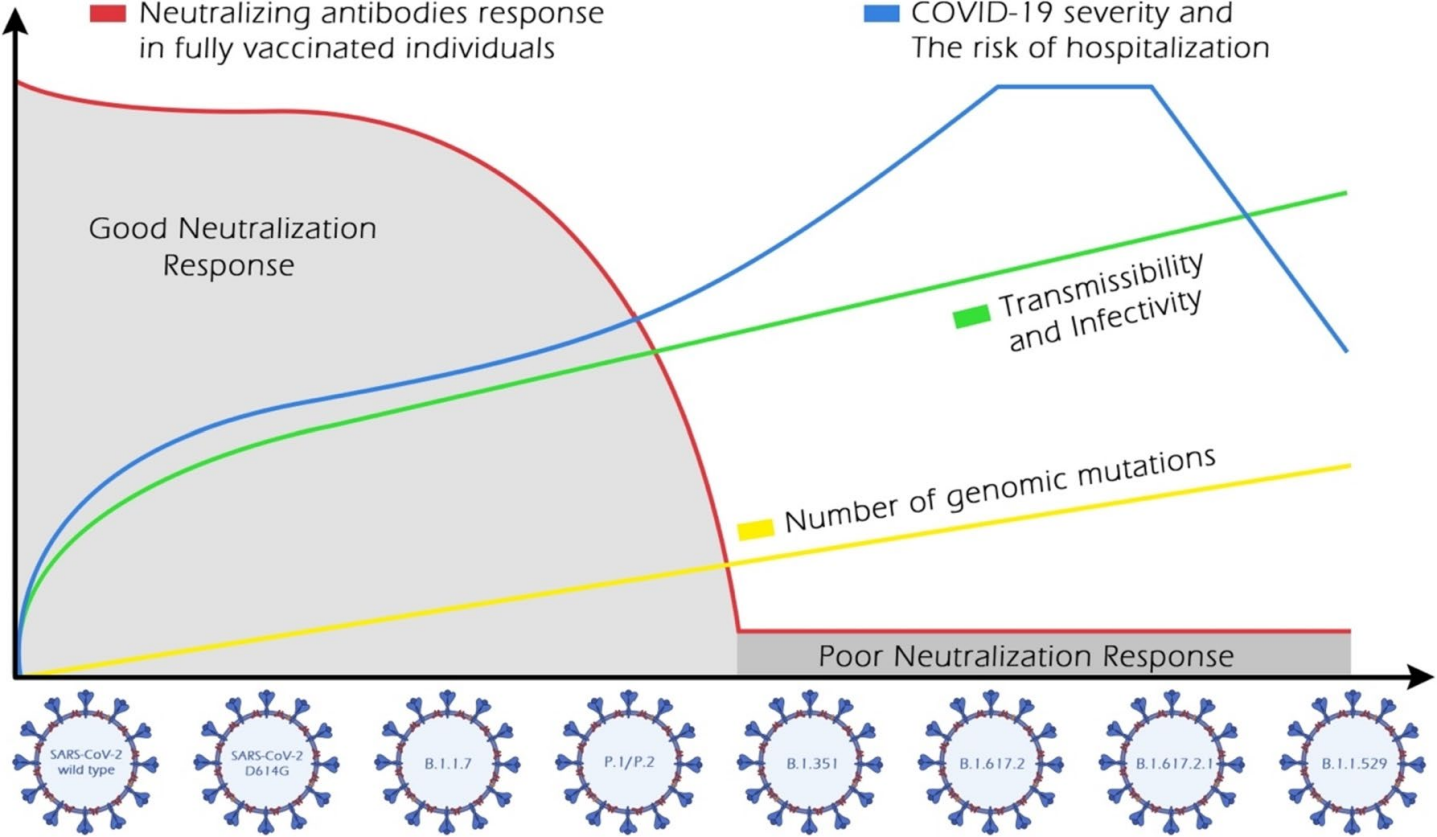
Schematic representation of the characteristics of the evolved SARS-CoV-2 phenotypes: the number of genomic mutations, transmissibility, and infectivity; the disease severity and the risk of hospitalization; the immunity escape

By April 2020, the D614G phenotype had begun to outcompete the ancestor SARS-CoV-2. In vitro and in vivo studies have revealed that the D614G variant is more contagious than the parental virus [22]. The contagiosity of D614G was explained by the substantial conformational changes that induce membrane fusion and virus internalization in the DG14G [55]. The emergence of the D614G variant was associated with an increase in viral load rather than an increase in disease severity and mortality rate. The effects of spike mutation, D614G, in SARS-CoV-2 on transmissibility and pathogenicity have been evaluated [21, 22]. A new wave of infections has surged worldwide due to the genetic evolution of the B.1.1.7 lineage. Scientific evidence revealed the higher transmissibility of B.1.1.7 than that of the preexisting variants [56]. Nevertheless, the higher global transmission was not accompanied by an increased COVID-19 severity rate, hospitalization, and deaths (Fig. 2) [23].

Growing evidence has reported the heightened transmissibility of B.1.351 lineage [57]. The increase in cases of B.1.351 infection and the associated disease burden (Fig. 2) provides substantial evidence of its ability to potentially evade the immunity acquired from infection and vaccination. Thus, patients previously exposed to other SARS-CoV-2 variants did not show cross-protection from re-infection with B.1.351 [25]. The rapid spread of P.1 and P.2 variants was documented through the increased number of hospitalized cases regionally and globally (Fig. 2) [30, 58]. The B.1.427/B.1.429 lineages have demonstrated rapid transmission in California, with transmissibility of 18–24% higher than that of the preexisting variants [33]. Unlike the N501Y mutation, L452R is not directly involved with ACE2 receptors. This mutation stabilizes spike-ACE2 interaction by inducing conformational changes in the spike. The transmissibility of variants carrying L429R and D614G is potentially higher than the phenotypes with D614G mutation alone but lower than those carrying N501Y mutation [15, 33, 38].

The emergence of the B.1.526 variant occurred while the pre-existing B.1.17 strain was dominant in New York, USA. Genomic surveillance revealed that the growth rate of B.1.526, particularly B.1.526-E484K, increased to 50%, while the frequency of other variants declined simultaneously. Moreover, B.1.526 led to a 35% higher hospitalization and transmission rate. Interestingly, due to the higher fitness of the B.1.1.7 variant, B.1.526 prevalence declined over time, with B.1.1.7 surpassed over time [35]. B.1.617.2 phenotype exhibits stronger transmissibility than other derivative variants. From October 2020 to March 2021, the Delta variant spread to over 200 countries worldwide [19, 59]. The viral virulence, disease severity, rate of hospitalization, admission to intensive care units, and mortality associated with Delta strains have been reported in various countries [24, 60] (Fig. 2). Interestingly, severe consequences have been reported in some patients infected with B.1.617.2 who were receiving anti-spike monoclonal antibodies (mAbs) therapies [61, 62]. Delta and Delta Plus are highly contagious strains, with transmissibility twice higher than the original Wuhan strain. Additionally, Delta Plus exhibits higher infectivity than the Delta variant (Fig. 2). The high transmission of Delta Plus could be attributed to the acquisition of K417N spike mutation, which plays a crucial role in the viral entry to the host cells through its interaction with ACE2 receptors [63].

The highly transmissible and infective Omicron BA.1 variant spread rapidly to more than 30 countries worldwide in less than two months (Fig. 2). The simultaneous presence of Y501N, Q498R, and S447N mutations in Omicron BA.1 synergistically enhances the spike-ACE2 binding [36]. Furthermore, the H505 mutation may further enhance the improved spike-ACE2 binding induced by these three mutations, concurrently [17]. Fortunately, Omicron BA.1 exhibits lower fusogenicity than Delta and the ancestral SARS-CoV-2 variants, resulting in reduced disease severity and low risk of hospitalization. Omicron sub-variants have demonstrated higher contagiousness and transmissibility than the parental Omicron BA.1 [64, 65].

In January 2022, several countries reported a surge of Omicron BA.2, which surpassed the predominant Omicron BA.1. Yamasoba et al. reported that the Omicron BA.2 spike exhibits higher reproducibility, fusogenicity, and pathogenicity than the BA.1 spike [66]. As of April 2022, Omicron BA.4 and BA.5 had surpassed the preceding Omicron sub-variants, BA.2. The higher transmissibility of Omicron BA.4 and BA.5 could be attributed to the ability of L452R-mutated spike to regulate ACE2 binding and evade humoral immunity acquired through infection or vaccination [67]. In May 2022, Omicron BA.2.75 emerged in India, which carries D339H, and N460K spike mutations, in addition to the L452R mutation, contributing to a higher ACE2 binding affinity and fusogenicity. Moreover, Omicron BA.2.75 exhibits comparable pathogenicity to Omicron BA.5 but higher than the sub-variant, BA.2 [49] (Fig. 2). The diverse mutations in the spike protein of XBB and its subvariants (XBB.1. and XBB.1.5) confer them with heightened transmissibility compared with other Omicron subvariants. Notably, the F486P mutation in XBB.1.5 has contributed to its enhanced infectivity and transmissibility [51]. Convergent mutations have resulted in varied binding affinity to the ACE2 receptor. Additionally, A484A to A484R, K444T to K444M, and F486S to F486P substitution mutations have resulted in enhanced affinity to the ACE2 [68]. Ito and his colleagues have demonstrated that R346T and N460K substitutions enhance the binding affinity of BQ.1.1 to ACE2, its infectivity, and fusogenicity [69].

## The antigenic impact of various SARS-CoV-2 variants

Neutralizing antibodies start to appear in the serum of COVID-19-infected individuals within ten days after the onset of symptoms and persist in circulation for a minimum of eight months [70, 71]. Over the two years of the SARS-CoV-2 pandemic, two mRNA-based vaccines, specifically Moderna (mRNA-1273) and Pfizer/BioNTek (BNT-126b2), were developed based on the spike glycoprotein isolated from the ancestral Wuhan strain. mRNA-based vaccines stimulate the host’s immune system to produce neutralizing antibodies that target the spike glycoprotein. Following the administration of the second booster dose, vaccine-induced antibodies are detectable in circulation for up to three months [72]. In addition to vaccines, the FDA has approved numerous mAbs for emergency use authorizations (EUA). Furthermore, several mAbs targeting the NTD of the spike glycoprotein and others targeting the RBD region are currently being investigated [73].

Neutralizing antibodies induced by prior SARS-CoV-2 infection or vaccination target specific epitopes in the NTD or RBD of the trimeric spike protein. Most of the anti-NTD antibodies interact with antigenic residues at 141–156 sites of the N3 loop or with supersites at 246–260 in the N5 loops [26, 28]. Genomic changes in the antigenic NTD supersites could affect the ability of the variants to evade humoral immunity. Anti-RBD neutralizing antibodies are the most widely utilized, abundant, and more potent than their anti-NTD counterparts [74]. Considering the diversity of neutralizing antibodies targeting the RBD, anti-RBD antibodies have been further classified into four classes based on their epitopes within the RBD [75]. Class I and II epitopes overlap with the ACE2-RBD binding sites, with class I specifically binding to RBD residue in the “up” conformation.

In contrast, class II antibodies interact with the RBD in the “up” or “down” conformation. Additionally, class III antibodies, known for their exceptional potency, do not directly interfere with ACE2 receptor binding sites. Instead, they interact with the opposite side of the RBD, including the loop region, in either “up” or “down” conformation. Class IV antibodies are the rarest and less potent, which target a cryptic epitope outside the RBM [75].

Researchers have established a comprehensive antibody escape map to investigate the antigenic impact of acquired mutations. As demonstrated by Greaney et al., class I antibodies bind to RBD residues K417, D420, N460, and A475. Consequently, mutations at any of these sites could diminish or nullify their virus-neutralization abilities. Genetic mutations at the E484, F490, and Q493 sites disrupt the neutralizing capabilities of class II antibodies. Mutation residues evade class III antibodies on the opposite side of RBD, encompassing R346, N440, K444, G446–N450, and Q498R. Class IV antibodies are interconnected with their RBD epitopes, either directly or indirectly. Mutations at RBD sites (369, 377, 378, or 384) enable the evasion of directly networked class IV antibodies, while combinatorial mutations, such as G339D, S371L, S373P, and S375F are likely to disrupt the binding of indirectly networked antibodies [76].

Specific neutralizing antibodies share epitope overlap at RBD binding sites. For instance, there is an overlap between class I and II antibodies at RBD sites, namely L455, F456, F486, and Y489. Certain mutations at RBD residue F490 also affect class II and III antibodies. Consequently, mutations that arise in the overlapping residues lead to antibody escape in both classes [76]. Cao and colleagues proposed an alternative escape map parallel to the one presented by Greaney et al., with minor variations [74, 76]. Cao et al. noted that genetic mutations at the supersites, K417, D420, F456, A475, and L455, often resulted in resistance to class A antibodies. The mutations at the F486, N487, and G476 sites escape the epitopes of class B antibodies [74].

Class C antibodies are the most potent among the antibody classes and are particularly sensitive to the antigenic E484 site. Additionally, class D antibodies target the RBD loop formed by 440–449 sites, whereby genomic changes in N440, K444, G446, and N448 sites result in the evasion of these antibodies [74]. Anti-RBD antibodies of class E and F are potentially less effective against various SARS-CoV-2 variants. Class E antibodies are affected by changes in G339, T345, and R346 sites, and some of these antibodies are sensitive to the N440 site at the RBD loop. Conversely, the epitopes of class F antibodies are regulated by mutations at F374, T376, and K378. In addition, mutations at V503 and G504 sites enable the evasion of certain class F antibodies, which may contribute to their potential ability to interact with ACE2 receptors [74].

Monoclonal antibodies including casirivimab (REGN10933), imdevimab (REGN10987), bamlanivimab (LY-CoV555), etesevimab (LY-CoV016), sotrovimab (VIR-7831), tixagevimab (AZD8895, the engineered mAb of COV2-2196), cilgavimab (AZD1061, engineered mAb of COV2-2130), and most recently, bebtelovimab (LY-CoV2-1404), have received EUA for mono- or dual therapy. Additionally, casirivimab (REGN10933), imdevimab (REGN10987), bamlanivimab (LY-CoV555), etesevimab (LY-CoV016), sotrovimab (VIR-7831), and bebtelovimab (LY-CoV2-1404) have been approved for the treatment of mild to moderate COVID-19 cases [76]. The FDA has also authorized the use of tixagevimab (AZD8895) and cilgavimab (AZD1061) as prophylactic interventions for immune-compromised patients with moderate-to-severe cases and for those whom approved vaccines are not recommended. FDA-authorized mAbs interact with their RBD epitopes either directly or indirectly. The neutralizing capabilities of the authorized therapeutic mAbs have also been mapped against their RBD epitopes. The indirect interaction between sotrovimab and spike residues at positions 339, 373, 440, and 446 has been reported. Casirivimab and imdevimab directly bind to RBD sites at 417, 440, 446, 484, 493, 496, and 498, whereas bamlanivimab and etesevimab directly interact with RBD residues at 417, 484, 493, 501, and 505 [76, 77]. According to Westendorf et al., bebtelovimab-RBD binding epitopes encompass residues at 345, 346, 4 417, 439–450, 452, and 498–506 (Table 3) [78].

**Table 3:**
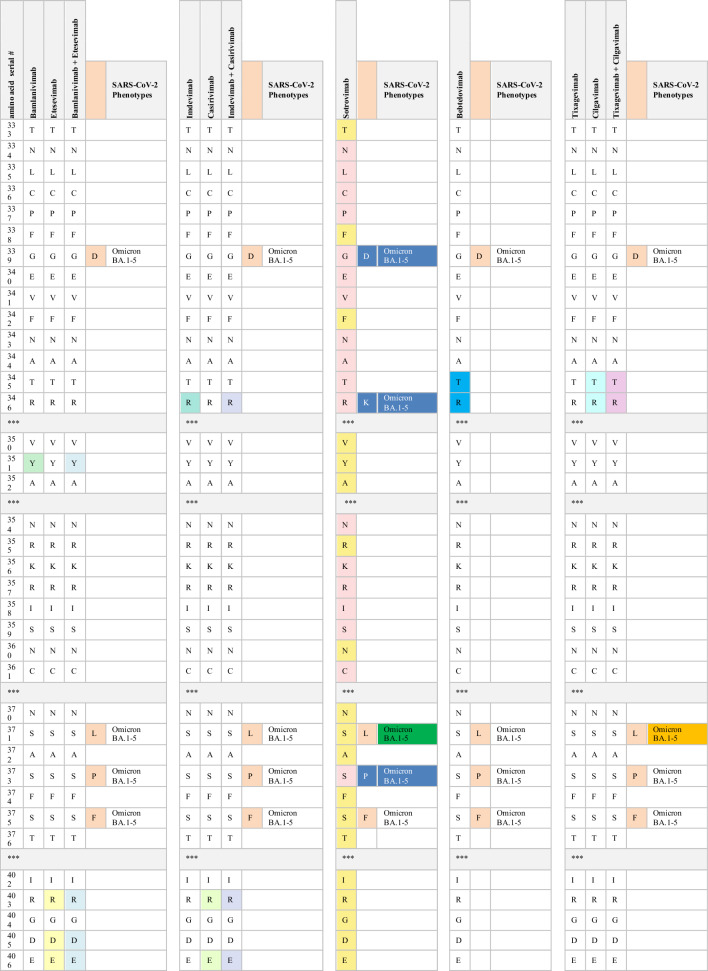

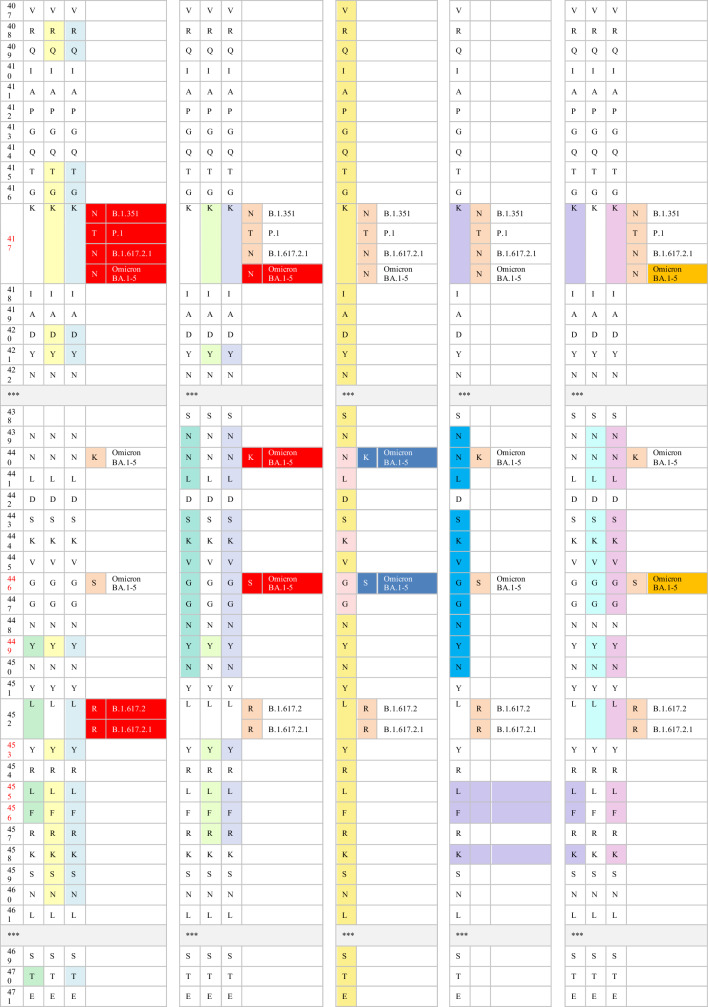

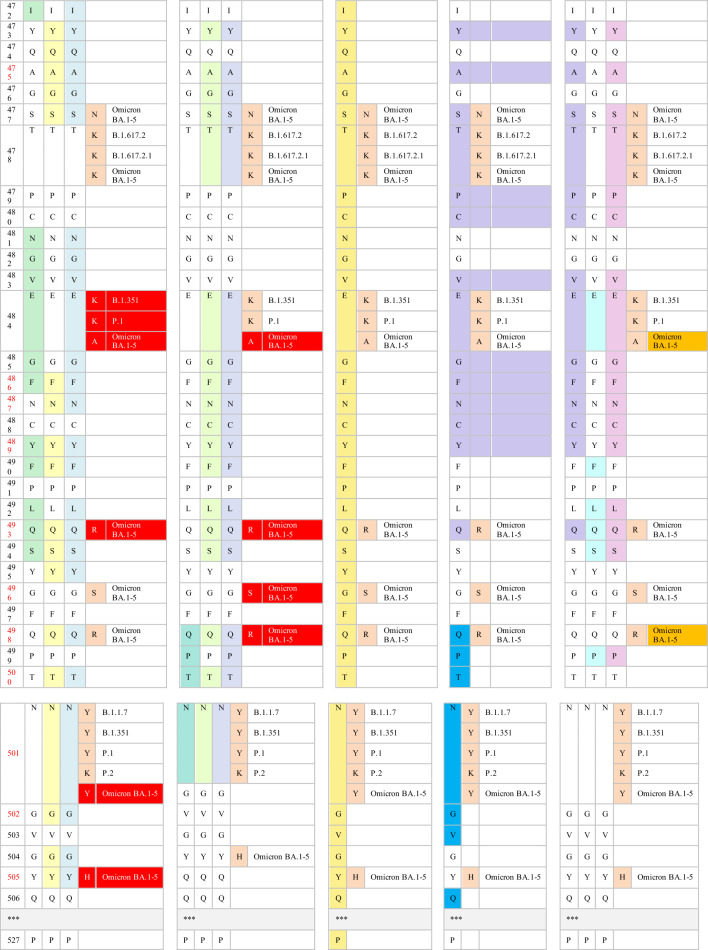

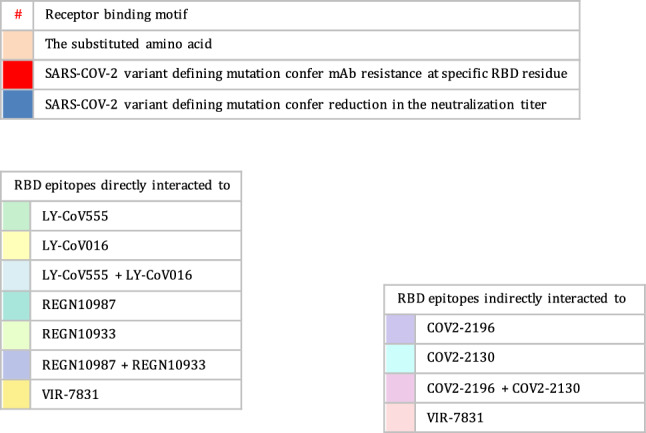
The neutralization titer of FDA EUA monoclonal antibodies against SARS-CoV-2 VOC

Multiple studies have demonstrated the inability of the variants carrying the D614G mutation to evade human immunity and develop resistance to spike-targeted immunological therapeutics. Since the crucial D614 mutation is located in the RBD residues, variants with the D614G mutation are effectively neutralized by antibodies induced by SARS-CoV-2 infection and vaccination [55, 79, 80].

Immunological studies have indicated that both convalescent sera and vaccines are generally effective in patients infected with the B.1.1.7 variant. In vitro studies have shown that the neutralization capacity of mRNA-vaccinated sera against B.1.1.7 declined after the first dose but not after the booster dose, in comparison with the reported efficacy against the original Wuhan strain. Also, single or combined mutations in B.1.1.7 do not significantly impact the activity of the vaccine-induced or pre-infection antibodies [80, 81]. The neutralizing activity of many antibodies targeting NTD and some directed to the RBD against B.1.1.7 variants significantly diminishes. The resistance in B.1.1.7 against anti-NTD and anti-RBD antibodies is attributable to the NTD supersite 144 del and the N501Y RBD mutation, respectively. Additionally, authorized mAbs have demonstrated effective cross-neutralization activity against SARS-CoV-2 B.1.1.7 (Table 3) except S309 (the parent of VIR-7831, sotrovimab), which exhibits a slight reduction in neutralizing activity [81, 82].

The cross-neutralization activity of convalescent and vaccine sera has significantly reduced when faced with emergent strains carrying mutations, such as E484K or the triad mutations (N501Y, K417N, and E484K). Gracia-Beltran et al. demonstrated a reduced neutralization capacity in mRNA-vaccinated individuals by B.1.351 variants, even after the booster dose [16]. The loss of activity in antibodies induced by vaccination and infection can be attributed to the critical E484K mutation within an immunodominant RBD epitope recognized by neutralizing antibodies [15, 83]. Furthermore, B.1.351 has shown a significant decline in susceptibility to neutralization by most anti-NTD and anti-RBD antibodies. The presence of multiple mutations in NTD supersites (242–244 del, R246I) and RBD residues (E484K, N417K) accounts for these findings [27]. Most notably, B.1.351 has exhibited resistance to several clinically used mAbs. The neutralizing activity of bamlanivimab and etesevimab, whether administered individually or in combination, is entirely abolished against the B.1.351 strain due to direct interactions with the E484K and N417K RBD mutations, respectively. Additionally, the significant decrease in the neutralizing activity of imdevimab against B.1.351 can be attributed to the presence of both the E484K and N417K mutations [81, 84, 85]. B.1.351 phenotypes have also shown partial resistance against the neutralizing activities of casirivimab. However, the combination of imdevimab and casirivimab retains their activity against B.1.351 [84]. While the potency of S309 is reduced to a certain degree, its neutralizing activity remains effective against B.1.351 (Table 3) [81, 83–85].

Similarly, the presence of triple RBD mutations, namely N501Y, K417T, and especially E484K in P.1 and P.2 variants, accounts for their notable ability to evade neutralizing antibodies produced in convalescent sera and through vaccinations [81, 86]. P.1 has exhibited resistance to neutralizing antibodies induced by vaccines comparable to the neutralization of B.1.1.7. However, its resistance is not as pronounced as the neutralizing capacity observed against the B.1.351 variant. The variability in neutralizing humoral antibody responses between P.1 and B.1.351 suggests that NTD mutations primarily confer resistance to convalescent sera or vaccine-induced antibodies [86, 87]. Conversely, mRNA-based vaccines provide partial cross-neutralization against P.1 and P.2 variants. A significant decline in the neutralizing antibody response has been reported in fully vaccinated individuals infected with P.1 or P.2 strains [16, 88]. These findings have been corroborated by numerous cases of re-infection with P.1 and P.2 variants [14, 31, 32]. Moreover, the P.1 lineage evades a multitude of EUA mAbs targeting RBD. Only imdevimab retains the ability to neutralize the P.1 variant, while the neutralizing capabilities of casirivimab, estevimab, and bamlanivimab have significantly diminished (Table 3). The resistance of P.1 against casirivimab and bamlanivimab may be attributed to their ability to partially or completely inhibit the entry of P.1 into host cells, respectively [84, 87]. Furthermore, the sensitivity of P.1 to anti-RBD antibodies, both under investigation and in clinical use, is comparable to that of B.1.351. However, the P.1 variant exhibits a distinct neutralizing pattern against NTD-targeting mAbs compared with B.1.351. This is explained by the diversification of NTD genomic mutations between these two strains, where L18F, T20N, D138Y, and R190S have been attributed to the resistance of many NTD-directed antibodies against P.1 but not against B.1.351 [87].

Phenotypes carrying the L452R mutation exhibit a relative resistance to antibodies generated by prior SARS-CoV-2 infection and mRNA vaccines [16, 33]. In-vitro studies have examined the impact of unique mutations within each B.1.526 sub-lineage on the response of neutralizing antibodies. Notably, the S477N mutation has produced no discernible antigenic effect. Consequently, resistance to B.1.526.2 infection has been reported in individuals who have recovered from COVID-19 and those who have been vaccinated. Mild to moderate symptoms in B.1.526.2-infected patients could also be treated with clinically EUA mAbs. Casirivimab and imdevimab maintain their neutralizing activities against B.1.526.2 when administered as monotherapy or in combination [89].

Conversely, the B.1.526-E484K lineage has exhibited a relatively reduced sensitivity to antibodies induced by convalescent sera and vaccination. The decrease in the neutralizing antibody response against this variant is similar to that observed against P.1 but less pronounced than B.1.351 [35, 90]. The B.1.526-E484K variant also exhibits resistance to monotherapy using several EUA mAbs, including casirivimab and bamlanivimab [35]. Nevertheless, the combined therapy of casirivimab and imdevimab has effectively neutralized the B.1.526-E484K variant [89]. Anti-RBD antibodies have also displayed notable variations in their neutralization potency against the B.1.526-E484K variant. Specifically, B.1.526-E484K demonstrates significant resistance to class II antibodies, in addition to a modest decline observed in the neutralizing response of class III [90].

A slight reduction in neutralization potency has been reported in the B.1.617.1 variant when exposed to convalescent plasma and vaccine sera. However, this decrease is not considered significant, suggesting that immunity conferred by mRNA-based vaccines remains effective against this variant [43, 91, 92]]. These results are comparable with the findings of Yadav et al., which confirmed the cross-neutralization of B.1.617 strains by the antibodies acquired from the infection and vaccine derived from BBV152 [42, 91, 92]. In their study, Hoffman et al. examined the antigenicity of the B.1.617 variant using therapeutic mAbs. Their findings demonstrate a minimal inhibitory effect of casirivimab on the entry of B.1.617 spike protein into host cells. The findings also indicate that the use of imdevimab, either alone or in combination with casirivimab, significantly reduces this entry. Another study reported a decrease in the effectiveness of bamlanivimab monotherapy or combination with etesevimab against SARS-CoV-2 B.1.617 [91]. Likewise, B.1.617.2 carrying L452R phenotypes are refractory for the humoral antibodies induced by vaccines and natural infection. Increased numbers of breakthrough infections by the Delta variant have been reported worldwide. Compared with the pre-existing variants, the susceptibility of partially or fully vaccinated individuals to the re-infection by the B.1.617.2 variant is more than 60%, while less than 5% was reported for B.1.1.7 and B.1.617.1 [93]. The increased infectivity of the B.1.617.2 variant can be attributed to its high viral load, which is approximately 1000 times higher than that of the original Wuhan isolates [94, 95]. Interestingly, individuals who were partially or fully vaccinated and contracted B.1.617.2 infection typically experience mild or asymptomatic cases [96]. Neutralization studies have also revealed the lower inhibitory effect of antibodies induced by the infection and two doses of sera-derived vaccines against B.1.617.2 than the B.1.1.7 variant.

Antibodies generated in individuals who received a single dose of vaccine exhibit negligible potency against the B.1.617.2 variant [96, 97]. In contrast, a potent neutralization of mRNA vaccines has been reported in vaccinated individuals after the third dose [98–100]. Furthermore, the resistance of B.1.617.2 against certain anti-NTD and anti-RBD mABs has been reported. Its resistance against anti-RBD antibodies can be primarily attributed to the presence of the L452R and T478K mutation [101]. Bamlanivimab was proven the least potent authorized mAbs against the Delta variant due to the resistance in L452R-mutated variants. Conversely, the effectiveness of casirivimab, etesevimab, and imdevimab against B.1.617.2 has not diminished (Table 3) [91, 97]. The Delta Plus variant has shown no discernible antigenic effect compared with the Delta variant. The magnitude of neutralizing antibodies response from previously infected sera and convalescent sera against Delta Plus is comparable with the response observed in the Delta variant [102]. Notably, the neutralizing capabilities of both bamlanivimab and etesevimab are completely lost against the Delta Plus variant [102]. The resistance of etesevimab is likely associated with the presence of the K417N mutation (Table 3) [103].

The Omicron BA.1 variant exhibits a remarkable ability to evade humoral immunity, primarily due to its extensive mutation profile. Moreover, it can escape the neutralizing antibodies from previous SARS-CoV-2 infections or vaccinations. Studies have shown that Omicron BA.1 completely resists the neutralization by mRNA-based vaccines in recently vaccinated individuals. However, booster doses of these vaccines have significantly enhanced neutralizing antibody responses against Omicron BA.1, with the third dose being particularly effective [98–100, 104].

Neutralization assays have revealed that most anti-RBD antibodies belonging to classes A-D experience a substantial reduction in their neutralizing titers against Omicron BA.1. This decline in neutralization efficacy can be attributed to the cumulative genetic mutations at various sites in the spike protein, including K417N, S477N, Q493R, G496S, Q498R, N501Y, and Y505H. These mutations collectively reduce the binding affinity between class A antibodies and ACE2 receptors. Additionally, mutations in Omicron BA.1, namely S477N, T478K, and E484A may enable the evasion of Class B antibodies. Despite differences in the amino acid substitution at the E484 residue between Omicron BA.1 and the B.1.351 variant, both mutations exhibit relatively comparable regulatory effects on the ability of the variants to evade immune responses. Mutations such as N440K and G446S in the RBD loop of Omicron BA.1 significantly reduce neutralization capabilities observed in class D antibodies [74].

Conversely, class E and F antibodies are more likely to retain their effectiveness against Omicron BA.1. Furthermore, the reduced neutralization of most anti-NTD antibodies against Omicron BA.1 can be attributed to deletions at positions 143–145 within the NTD [74]. In line with findings from Cao et al., Miller et al. reported the cumulative mutations in the Omicron BA.1 variant, which resulted in a broader range of antibody escape (Classes I-IV) and greater depth of escape (Class I) than the pre-existing variants, such as B.1.351 and Delta. This heightened escape capacity poses significant challenges to neutralizing antibody responses [17]. Moreover, emerging data highlight the capability of the Omicron BA.1 variant to escape the neutralization potency of authorized mAbs. The extensive mutations present in Omicron BA.1, particularly within the RBM of the spike protein, result in a loss of neutralizing activity of several mAbs.

Neutralizing activities of casirivimab, imdevimab, bamlanivimab, and etesevimab, whether administered as monotherapy or in combination therapy, are completely abrogated against Omicron BA.1. This loss of efficacy is primarily attributed to the cumulative mutations at residues 417, 440, 446, 484, 493, 496, 498, 501, and 505 within their spike proteins. Consequently, the US FDA has recently imposed restrictions on the use of previously authorized mAbs, such as casirivimab, imdevimab, bamlanivimab, and etesevimab, in the treatment of Omicron-infected patients. These mAbs are now recommended only for individuals exposed to or at high risk of infection with mAbs-susceptible variants [105]. The monotherapy using tixagevimab and cilgavimab has exhibited weak neutralizing activities against Omicron BA.1. Resistance to tixagevimab is primarily attributed to the spike mutations, namely S371L/F, K417N, E484A, and Q498R. Additionally, the insensitivity toward cilgavimab is conferred by G446S and E484A mutations. However, the combined therapy of tixagevimab and cilgavimab synergistically enhances their effectiveness against Omicron BA.1 [106].

In February 2022, the FDA recommended doubling the initial preventive dose of tixagevimab and cilgavimab to 300 mg each (instead of 150 mg each) to enhance their effectiveness against Omicron sub-variants [107]. In contrast, indirect networking of mAbs has resulted in a moderate decrease in their neutralizing titer against Omicron despite retaining their activity (Table 3) [17, 108]. The susceptibility to the re-infection with Omicron BA.1.1 and BA.2 has been reported in individuals previously infected with preSARS-CoV-2 variants (Wild, Alpha, and Delta) and those who had received two mRNA vaccine doses. Three doses of mRNA-based vaccine generally offer moderate protection against Omicron BA.2 and BA.3 infections [109, 110]. Meanwhile, Omicron BA.2 evades neutralizing antibodies in unvaccinated or partially vaccinated individuals who had previously been infected with Omicron BA.1. However, fully vaccinated individuals previously infected with Omicron BA.1 are ultimately resistant to Omicron BA.2 infection [66]. Li et al. demonstrated a comparable antigenic behavior in Omicron sub-variants BA.3 against various SARS-CoV-2 spike mutations compared with Omicron BA.1 and BA.2. Additionally, Omicron BA.3 could efficiently evade neutralizing antibodies in individuals previously infected with D614G, Alpha, and Delta variants, and to a lesser extent, in those infected with Beta and Gamma variants, while remaining sensitive to antibodies in individuals infected with Omicron BA.1 [111].

The resistance toward neutralizing activities of casirivimab, imdevimab, bamlanivimab, etesevimab, and tixagevimab has been reported in Omicron BA.2 and BA.3 sub-variants. Omicron Compared to BA.1 sub-variant, BA.2 is less resistant to cilgavimab, making the combination of cilgavimab and tixagevimab more effective against BA.2. Furthermore, the absence of the G446S spike mutation in Omicron BA.2 enhances its sensitivity to cilgavimab [66, 111]. Similarly, Omicron BA.1 and BA.1.1 sub-variants can be neutralized by sotrovimab, whereas the RBD mutation (S371F) confers the resistance against sotrovimab to Omicron BA.2 [66].

The substitution of F486V in Omicron BA.4 and BA.5 sub-variants has reduced their susceptibility to RBD-targeted antibodies induced by prior infection or vaccination. This change diminishes their sensitivity to most class I and some class II monoclonal antibodies (mAbs). As with other SARS-CoV-2 variants carrying the L452R mutation, Omicron BA.4 and BA.5 exhibit a notable resistance to class II mAbs [48]. These sub-variants also resist authorized mAbs, including bamlanivimab, casirivimab, etesevimab, imdevimab, and tixagevimab. Compared to the BA.2 sub-lineage, which shares the L452R/Q mutations, Omicron BA.4 and BA.5 exhibit lower resistance to sotrovimab but higher resistance to cilgavimab and the combination of cilgavimab and tixagevimab. The newly approved mAb, bebtelovimab, remains effective against all Omicron sub-lineages, including BA.1, BA.2, BA.3, BA.4, and BA.5 [18].

It is worth noting that there is currently limited data on the sensitivity or resistance of Omicron BA.2.75 to therapeutic mAbs or humoral antibodies induced by natural infection or immunization. Preliminary studies have indicated that Omicron BA.2.75 is insensitive to sera from BA.2 and BA.5-infected hamsters. The capability of Omicron BA.2.75 to escape from neutralizing antibodies in BA.2-induced immunity is believed to be due to G446S mutation. Meanwhile, the resistance in Omicron BA.2.75 against BA.5-infected hamster sera has been attributed to K147E, W152R, F157L, and G446S mutations [49]. Gruell reported the resistance in Omicron BA.2.75 to bamlanivimab, casirivimab, etesevimab, and imdevimab while remaining susceptible to sotrovimab and tixagevimab. Additionally, the neutralizing sensitivity of BA.2.75 to cilgavimab is lower than that of BA.2. Omicron BA.2.75 also exhibits higher resistance against bebtelovimab than BA.2, BA.4, and BA.5 [112].

Research has suggested that the recombinant SARS-CoV-2 is the most escapist sub-lineage. The distinguishable mutations in these variants have resulted in the compromised efficacy of the commercially available vaccines and the neutralizing and mAbc antibodies. Studies have reported that R346T and N460K mutations confer antibody resistance to the target spike proteins. Additionally, variants with N460K and F486S mutations have shown resistance to the classes I and II mAbs, whereas F490S, R346T, G446S, and V455P mutations are associated with the resistance to the class III mAbs of RBD. Furthermore, V445P and K444T mutations increase the steric hindrance, which leads to the escape from the neutralizing and mAbs antibodies. A study reported the enhanced ACE-binding affinity in XBB.1.5 conferred by F486P mutation, which is manifested in its immune evasion properties [17]. Several studies have also demonstrated a superior evasion of humoral immunity induced by either natural infection or vaccination with monovalent mRNA vaccines in XBB and XBB.1 [113]. The currently developed bivalent booster vaccine has shown good protection against the newly emerged recombinant sub-lineages [114].

The neutralizing capacity of the convergent mutations against the therapeutic mAbs has been extensively investigated. More than a 100-fold reduction in IC50 of cilgavimab was reported in variants containing the spike mutation, R346I, compared with the wild mutation, R346K. Conversely, a study showed that R346T, R346R, and R346S mutations result in less than five reductions in the neutralizing capabilities of other therapeutic mAbs [68]. Omicron sub-lineages harboring different substitutions at K444 in the spike exhibit resistance to various mAbs, i.e., K444M and K444N are resistant to imdevimab; K444T is resistant to cilgavimab; K444R is resistant to imdevimab and bebtelovimab [68]. A study confirmed that the immune-evading capability of BQ.1.1 for the breakthrough of BA.2 and BA.5 infection sera is attributed to the R346T, K444T, and N460K RBD mutations [69]. Given the increasing genetic mutations causing antigenic shifts in the evolving Omicron sub-lineages, it is crucial to focus scientific attention on their potential global health risks.

## Conclusion and future outlooks

Given the ongoing evolution of new SARS-CoV-2 variants, it is clear that COVID-19 will continue to have far-reaching global health, social, and economic impacts. While numerous vaccines have been deployed to prevent SARS-CoV-2 infection, and several immunological therapies have been authorized for treating COVID-19 patients, we are in a race against time to keep pace with the emergence of antigenically distinct SARS-CoV-2 variants that carry critical mutations in the spike protein. The increasing incidence of amino acid substitutions and deletions in the spike protein has been linked to differences in transmissibility, infectivity, disease severity, and the effectiveness of neutralizing antibody responses. Predicting the emergence of specific SARS-CoV-2 mutations in the future is challenging. However, robust genomic sequencing of highly contagious variants provides essential information about the nature, extent, and immunological implications of SARS-CoV-2's antigenic drift.

Advanced antibody-escape maps serve as a valuable guide for anticipating the potential resistance to neutralizing antibodies induced by natural infections and vaccination and the efficacy of authorized mAbs against newly emerging variants. Early detection and monitoring of emerging antigenic phenotypes and rapid clinical investigations are critical for developing the next generation of vaccines and antibody-based therapies with broad cross-reactivity against the prevailing variants.

In conclusion, ongoing vigilance and research are essential in the fight against SARS-CoV-2 and its variants. Continuous efforts to understand and adapt to the evolving viral landscape will be crucial in controlling the impact of COVID-19 on a global scale.
