# Correction: SARS-CoV-2 journey: from alpha variant to omicron and its sub-variants

**DOI:** 10.1007/s15010-024-02283-0

**Published:** 2024-04-29

**Authors:** Dima Hattab, Mumen F. A. Amer, Zina M. Al-Alami, Athirah Bakhtiar

**Affiliations:** 1 School of Pharmacy, The University of Jordan, Queen Rania Street, Amman, Jordan; 2 Faculty of Pharmacy, Applied Science Private University, Amman, Jordan; 3 Department of Basic Medical Sciences, Faculty of Allied Medical Sciences, Al-Ahliyya Amman University, Amman, Jordan; 4 School of Pharmacy, Monash University Malaysia, Jalan Lagoon Selatan, 47500 Bandar Sunway, Selangor Malaysia

**Correction: Infection** 10.1007/s15010-024-02223-y

In this article the affiliation details for author.

Zina M. Al Alami^3,4^ are not correct.

^3^Department of Basic Medical Sciences, Faculty of Allied Medical Sciences, Al-Ahliyya Amman University, Amman, Jordan

^4^School of Pharmacy, Monash University Malaysia, Jalan Lagoon Selatan, 47,500 Bandar Sunway, Selangor, Malaysia

The correct details are given here:

Zina M. Al Alami^3^

^3^Department of Basic Medical Sciences, Faculty of Allied Medical Sciences, Al-Ahliyya Amman University, Amman, Jordan

The Original Article has been corrected.

